# Supplementary material for: Genetic screening identifies a SUMO protease dynamically maintaining centromeric chromatin
Source: Nat Commun. 2020 Jan 24;11:501. doi: 10.1038/s41467-019-14276-x (PMC6981222; doi:10.1038/s41467-019-14276-x)
Supplement: Supplementary file 1 — Supplementary Information [file 41467_2019_14276_MOESM1_ESM.pdf]

Supplementary Information

GENETIC SCREENING IDENTIFIES A SUMO PROTEASE DYNAMICALLY MAINTAINING CENTROMERIC CHROMATIN

Sreyoshi Mitra, Dani L. Bodor, Ana F. David, Izma Abdul-Zani, João F. Mata, Beate Neumann, Sabine Reither, Christian Tischer and Lars E.T. Jansen

5 supplementary figures with corresponding legends

Supplementary Figure 1

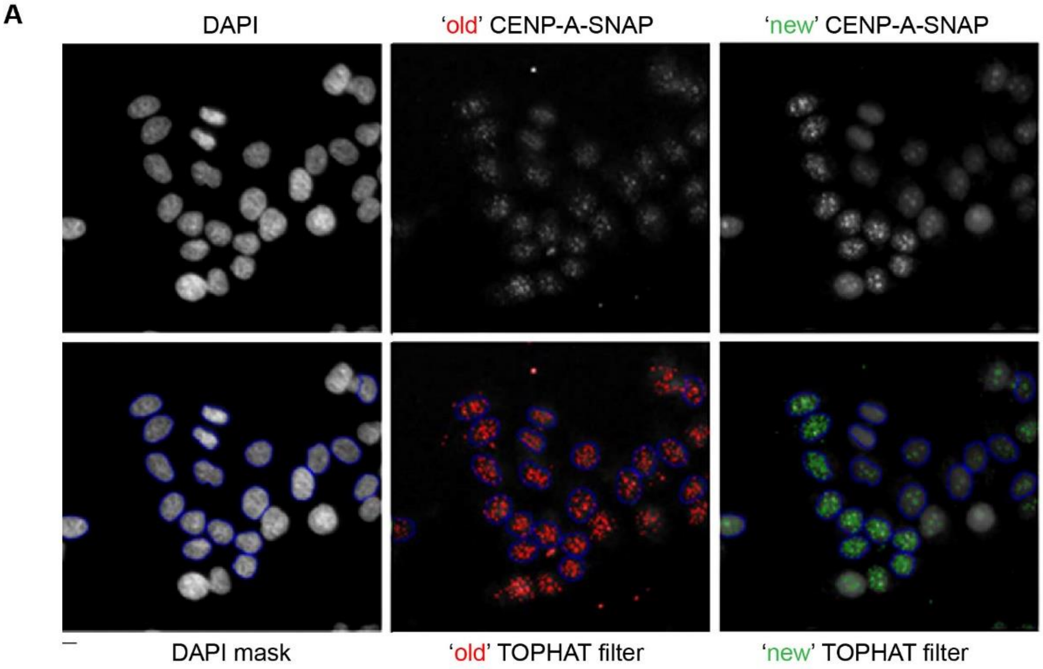

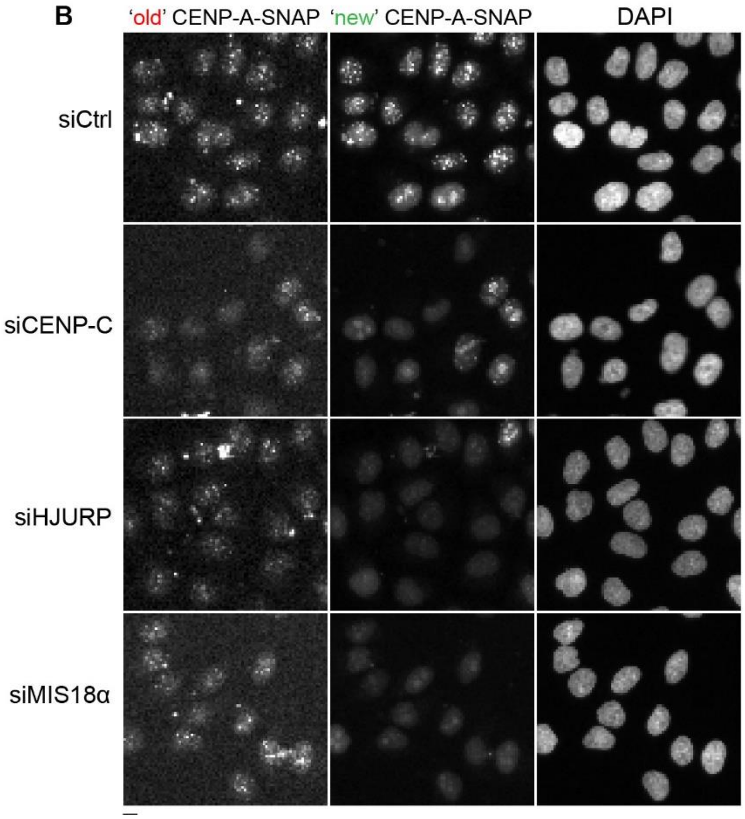

Supplementary Figure 1. Quantification of siRNA screen results by Cell Profiler pipeline. (A) Representative low magnification images depicting the various steps of the custom Cell Profiler pipeline used to quantify 'old' and 'new' CENP-SNAP fluorescent signals in the primary siRNA screen. A DAPI mask was

applied in order to identify interphase nuclei. Within the DAPI mask, custom TOPHAT filters were applied based on signal area and intensity in order to identify centromeres and measure 'old' and 'new' CENP-A-SNAP signal intensities. Bars, 10 µm. **(B)** Representative images for 'old' (siCENP-C) and 'new' (siHJURP, siMis18α) CENP-A-SNAP phenotypes used as control siRNAs in the screen. Bars, 10 µm.

Supplementary Figure 2

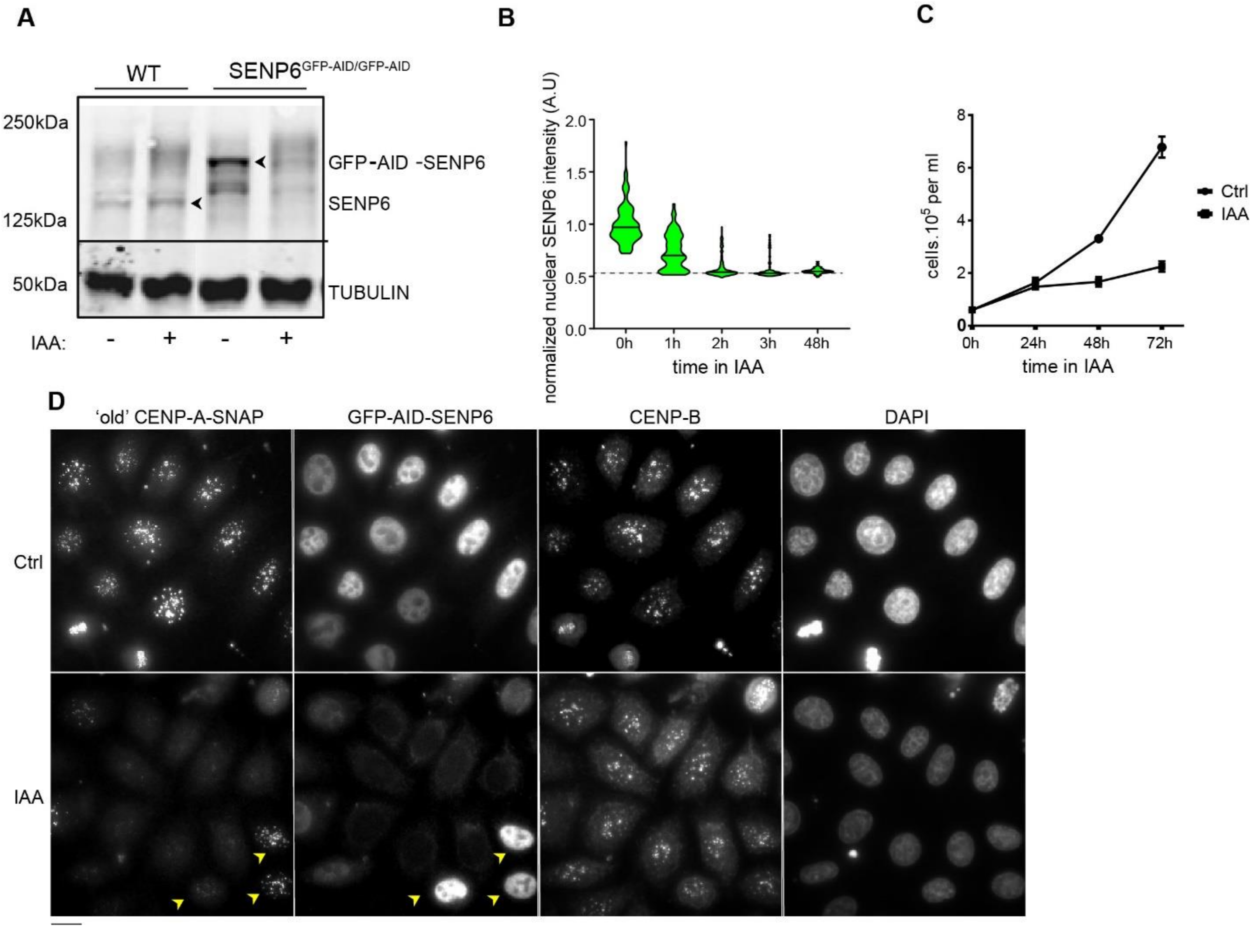

Supplementary Figure 2. Kinetics of CENP-A loss and growth arrest upon acute SENP6 loss. (A) Immunoblot showing the degradation of GFP-AID-SENP6 protein upon addition of auxin (IAA) in a homozygously tagged (SENP6 $^{GFP\text{-}AID/GFP\text{-}AID}$) cell line. Untagged wild-type (WT) and SENP6 $^{GFP\text{-}AID/GFP\text{-}AID}$ cell lines were treated with auxin or mock-treated for 24 hours, extracts were separated by SDS-PAGE and immunoblotted with anti-SENP6 antibody. Tubulin was used as the loading control. Arrowheads indicate the WT and tagged SENP6 respectively. **(B)** Kinetics of auxin (IAA)-mediated degradation of GFP-AID-SENP6 measured by fluorescence microscopy and normalized to the mean intensity at 0 hour time point is plotted as violins. At least 200 nuclei were measured under each condition. Bar indicates the median value. Dotted line indicates nuclear background fluorescence. **(C)** Long term auxin (IAA)-mediated depletion of SENP6 leads to cell division arrest. Cell numbers were measured as a function of time under auxin (IAA) treatment or mock-treatment (Ctrl). Two replicate experiments were performed. Bars indicate SEM. **(D)** Images of centromeric levels of pre-incorporated 'old' CENP-A-SNAP in a pulse-chase assay following auxin (IAA)-mediated depletion of GFP-AID-SENP6 for 24 hours as quantified in Figure 3C. Cells were counterstained with CENP-B as centromeric reference. Yellow arrowheads indicate nuclei which retain GFP-AID-SENP6 after auxin (IAA) treatment and correspondingly retain 'old' CENP-A-SNAP. Bar, 10 µm. Source data are provided as a Source Data file.

Supplementary Figure 3

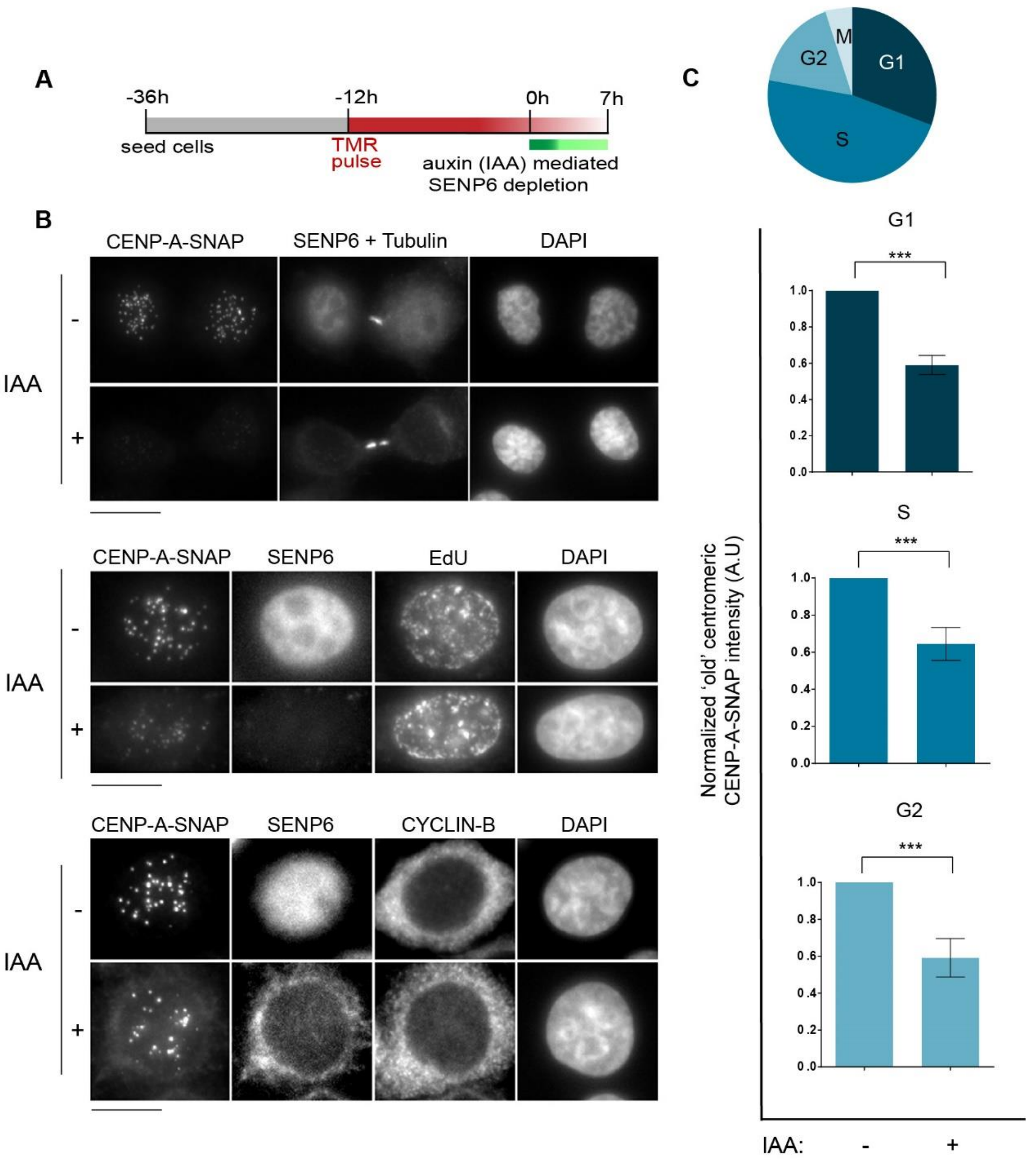

Supplementary Figure 3. SENP6 is required for maintaining CENP-A at all cell cycle stages. (A) Experimental scheme of CENP-A-SNAP pulse-chase assay following auxin (IAA) mediated depletion of SENP6 in unsynchronized cells. **(B)** Results of the experiment described in (A) showing images of centromeric levels of 'old' CENP-A-SNAP following auxin (IAA) mediated depletion of SENP6 at specific cell cycle stages. Cells were counter-stained with α-tubulin to score for mid-body positive G1 cells, EdU to score for S phase cells and with Cyclin B to stain G2 cells. Bars, 10 μm. **(C)** (Top panel) Schematic depicting relevant stages of the cell cycle. (Bottom panel) Quantification of (B). Centromeric CENP-A-SNAP signal intensities in cells under auxin (IAA) treatment (+) were normalized to the control mock-treated condition (-) in each experiment and plotted along the y-axis. Three replicate experiments were performed. Bars indicate SEM. Parametric two-tailed Student's t

test was performed to calculate statistical significance. ***: p<0.001. Source data are provided as a Source Data file.

Supplementary Figure 4

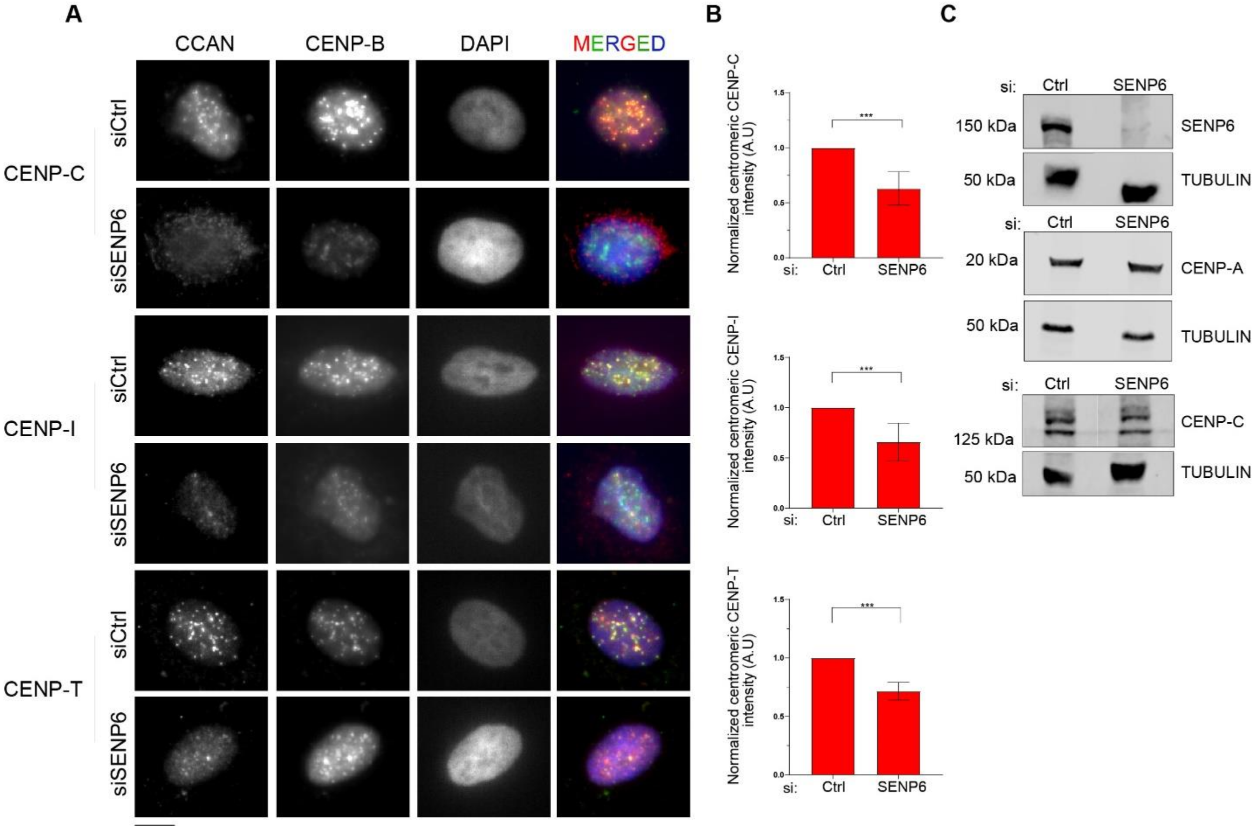

Supplementary Figure 4. SENP6 is required for CCAN integrity independent of proteolysis in untagged HeLa cells. (A) Centromeric levels of representative CCAN proteins following siRNA mediated depletion of SENP6 for 48h. Cells were counterstained with CENP-B to mark centromeres. Bars, 10 µm. **(B)** Automated centromere recognition and quantification of (A). Fluorescence intensities of indicated proteins were normalized to the mean of the control siRNA treated condition in each experiment and plotted as a bar graph following siRNA of SENP6 or scrambled Ctrl. Three replicate experiments were performed. Bars represent SEM. Parametric two-tailed Student's t test were performed to calculate statistical significance. ***: p<0.001. **(C)** Immunoblot showing the total levels of CENP-A and CENP-C proteins following 48 hour treatment with siRNA to deplete SENP6. Extracts from control or siSENP6 cells were separated by SDS-PAGE and immunoblotted with anti-CENP-A or anti-CENP-C antibody. Tubulin was used as loading control. Source data are provided as a Source Data file.

Supplementary Figure 5

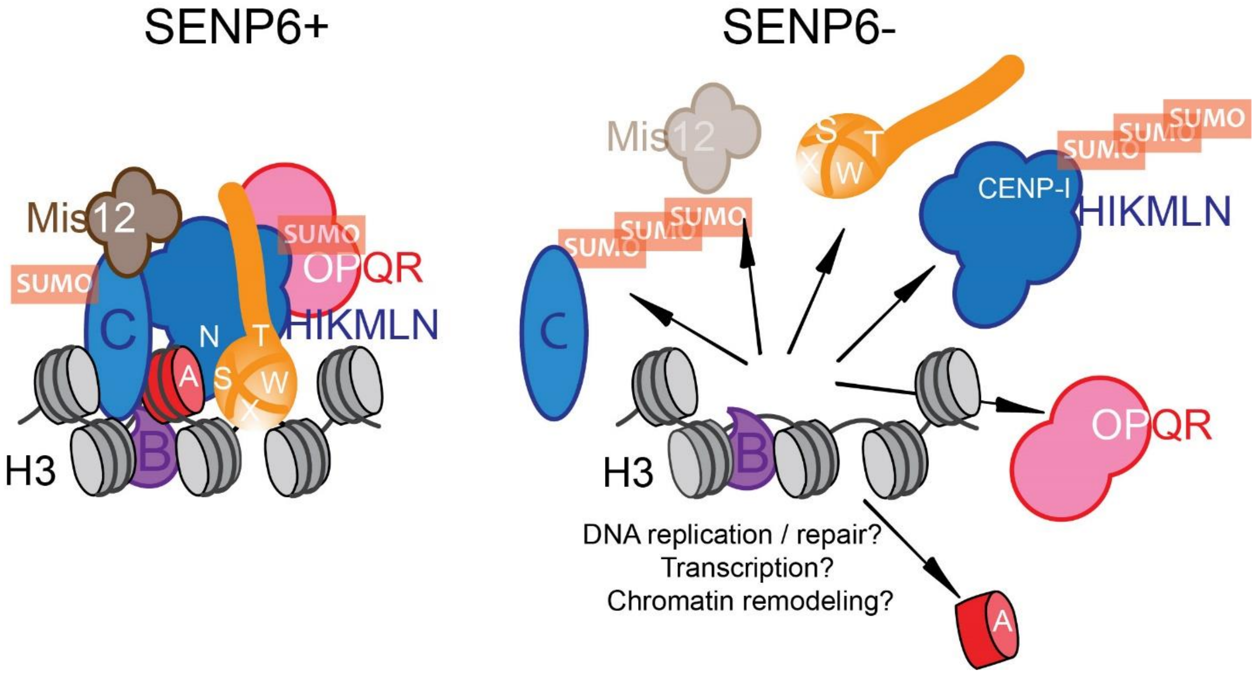

Supplementary Figure 5. Model illustrating the role of SENP6 in maintaining the centromere complex. SENP6 actively deSUMOylates key CCAN proteins to keep the inner kinetochore in a hypoSUMOylated and thereby intact state. In absence of SENP6 multiple CCAN proteins are polySUMOylated resulting in loss of kinetochore integrity and the resultant eviction of CENP-A from the centromere by, as of yet, unknown mechanisms.
