# Supplementary material for: Genetic screening identifies a SUMO protease dynamically maintaining centromeric chromatin
Source: Nat Commun. 2020 Jan 24;11:501. doi: 10.1038/s41467-019-14276-x (PMC6981222; doi:10.1038/s41467-019-14276-x)
Supplement: Supplementary file 3 — Reporting Summary [file 41467_2019_14276_MOESM3_ESM.pdf]

nature research

Corresponding author(s): Lars Jansen
Last updated by author(s): Dec 16, 2019

Reporting Summary

Nature Research wishes to improve the reproducibility of the work that we publish. This form provides structure for consistency and transparency in reporting. For further information on Nature Research policies, see Authors & Referees and the Editorial Policy Checklist.

Statistics

For all statistical analyses, confirm that the following items are present in the figure legend, table legend, main text, or Methods section.

n/a	Confirmed	
☐	☒	The exact sample size (*n*) for each experimental group/condition, given as a discrete number and unit of measurement
☐	☒	A statement on whether measurements were taken from distinct samples or whether the same sample was measured repeatedly
☐	☒	The statistical test(s) used AND whether they are one- or two-sided<br>*Only common tests should be described solely by name; describe more complex techniques in the Methods section.*
☐	☒	A description of all covariates tested
☒	☐	A description of any assumptions or corrections, such as tests of normality and adjustment for multiple comparisons
☐	☒	A full description of the statistical parameters including central tendency (e.g. means) or other basic estimates (e.g. regression coefficient) AND variation (e.g. standard deviation) or associated estimates of uncertainty (e.g. confidence intervals)
☐	☒	For null hypothesis testing, the test statistic (e.g. *F*, *t*, *r*) with confidence intervals, effect sizes, degrees of freedom and *P* value noted<br>*Give P values as exact values whenever suitable.*
☒	☐	For Bayesian analysis, information on the choice of priors and Markov chain Monte Carlo settings
☒	☐	For hierarchical and complex designs, identification of the appropriate level for tests and full reporting of outcomes
☒	☐	Estimates of effect sizes (e.g. Cohen's *d*, Pearson's *r*), indicating how they were calculated

Our web collection on statistics for biologists contains articles on many of the points above.

Software and code

Policy information about availability of computer code

Data collection	No software was used
Data analysis	Image analysis of the primary screen was performed using a CellProfiler (Carpenter et al., 2006) pipeline, available for download (https://git.embl.de/grp-almf/sreyoshi-mitra-jensen-centromere-screen/blob/master/publication/mitra_centromere_screen_cp2.2.0.cpproj) and can be used with CellProfiler version 2.2.0. Visual inspection, quality control and statistical analysis of the primary screen was performed using HTM explorer (https://github.com/tischi/HTM_Explorer). Graphs were plotted with GraphPad software.

For manuscripts utilizing custom algorithms or software that are central to the research but not yet described in published literature, software must be made available to editors/reviewers. We strongly encourage code deposition in a community repository (e.g. GitHub). See the Nature Research guidelines for submitting code & software for further information.

Data

Policy information about availability of data

All manuscripts must include a data availability statement. This statement should provide the following information, where applicable:
- Accession codes, unique identifiers, or web links for publicly available datasets
- A list of figures that have associated raw data
- A description of any restrictions on data availability

The raw data for Figure 1C and 1D has been provided in Supplemental tables 2 and 3 respectively.

Field-specific reporting

Please select the one below that is the best fit for your research. If you are not sure, read the appropriate sections before making your selection.

☒ Life sciences ☐ Behavioural & social sciences ☐ Ecological, evolutionary & environmental sciences

For a reference copy of the document with all sections, see nature.com/documents/nr-reporting-summary-flat.pdf

Life sciences study design

All studies must disclose on these points even when the disclosure is negative.

Sample size	For the primary siRNA screen (Figure 1), 1.5 x 10^5 cells were seeded onto the Labtek chamber so that after 48 hours there were on an average 100 cells per siRNA spot. This number was chosen by assessment from our pilot screens, which showed that centromeric fluorescence intensities from the above number of cells were sufficient to show the difference in CENP-A levels between our scrambled negative control siRNA treatment and the positive control siRNA treatments. For the validation experiments and the subsequent microscopy experiments in the paper, on an average 50 cells were imaged per condition in each experiment resulting in measurement of fluroscence intensities from 500 centromeres on an average. These numbers were based on CENP-A-SNAP based measurement experiments which have been reported in previous papers from our lab (Bodor et al., 2012; Stankovic et al., 2017).
Data exclusions	No data were excluded from analyses
Replication	For Figure 1C and D the experiments were repeated five times in different time frames constituting five full biological replicates. For Figures 2C-I, 3C-J, 4C, F,H, 5D, S3 and S4 the experiments were repeated three times in different time frames constituting three biological replicates. For Figure S2C the experiment to determine the growth delay was repeated twice.
Randomization	For the primary screen HeLa cells expressing CENP-A-SNAP were seeded in Labtek chambers printed with siRNA spots representing a custom siRNA library for 1046 genes. This setup also contained randomly positioned spots of a negative scrambled control siRNA as well as spots of siRNAs against known factors affecting CENP-A maintenance and/or assembly which serve as positive controls. For the validation siRNA experiments, similarly a negative scrambled siRNA control was always used. For the auxin based assays, the comparisons of CENP-A intensity were always made against a mock-treated control condition.
Blinding	During data collection coded identifiers were used and all fluorescent intensity measurements were conducted using an automated unbiased algorithm.

Reporting for specific materials, systems and methods

We require information from authors about some types of materials, experimental systems and methods used in many studies. Here, indicate whether each material, system or method listed is relevant to your study. If you are not sure if a list item applies to your research, read the appropriate section before selecting a response.

Materials & experimental systems

n/a	Involved in the study
☐	☒ Antibodies
☐	☒ Eukaryotic cell lines
☒	☐ Palaeontology
☒	☐ Animals and other organisms
☒	☐ Human research participants
☐	☐ Clinical data

Methods

n/a	Involved in the study
☒	☐ ChIP-seq
☒	☐ Flow cytometry
☒	☐ MRI-based neuroimaging

Antibodies

Antibodies used	The following antibodies were used in the manuscript: mouse monoclonal anti-CENP-A [gift from Kinya Yoda (Nagoya University) (Ando et al., 2002)]; rabbit polyclonal anti-CENP-B (sc22788; Santa Cruz Biotechnology, Dallas, TX); rabbit polyclonal anti-CENP-B (ab25734; Abcam) at 1:500; mouse monoclonal anti-CENP-B (ab167361; Abcam), mouse monoclonal anti-CENP-C isolated from hybridoma line (LX191) [gift from Don Cleveland, UCSD (Foltz et al., 2009)], rabbit polyclonal anti CENP-T [gift from Don Cleveland, UCSD (described in (Barnhart et al., 2011); rat monoclonal anti-CENP-H and rabbit polyclonal anti-CENP-I (both gifts from Song-Tao Liu, University of Toledo); rabbit polyclonal anti-DSN1 (gift from Iain Cheeseman, Whitehead) rabbit polyclonal anti-NNF1 (gift from Arshad Desai, UCSD); mouse monoclonal anti-HEC1 (Thermo Scientific Pierce MA1-23308), sheep polyclonal anti-SENP6 (gift from Ronald Hay, Dundee), rat monoclonal anti-Tubulin (SC-53029, Santa Cruz Biotechnology, Dallas, TX) and mouse monoclonal anti Cyclin-B (SC-245, Santa Cruz Biotechnology, Dallas, TX)
Validation	All the primary antibodies used in the manuscript have been used in previous papers as cited above or validated primary antibodies from commercial vendors as mentioned above.

Eukaryotic cell lines

Policy information about cell lines

Cell line source(s)	The parent cell line used was unmodified HeLa cells or HeLa-CENP-A-SNAP clone #72 as described in (Jansen et al., 2007; Bodor et al., 2012). Based on this parent, a cell line was constructed in this paper which expressed OsTir1 as a trans-gene and the SENP6 gene was homozygously tagged with GFP-AID construct at its endogenous locus. Primary human cells were derived from healthy fetal skin fibroblast (obtained from Coriell Cell Repository #GM06170).
Authentication	The GFP-AID-SENP6 cell line was authenticated for homozygous tagging of the SENP6 gene by immunoblot using sheep anti-SENP6 antibody.
Mycoplasma contamination	All cell lines were tested and scored negative for mycoplasma contamination.
Commonly misidentified lines (See ICLAC register)	*Name any commonly misidentified cell lines used in the study and provide a rationale for their use.*

Clinical data

Policy information about clinical studies

All manuscripts should comply with the ICMJE guidelines for publication of clinical research and a completed CONSORT checklist must be included with all submissions.

Clinical trial registration	*Provide the trial registration number from ClinicalTrials.gov or an equivalent agency.*
Study protocol	*Note where the full trial protocol can be accessed OR if not available, explain why.*
Data collection	*Describe the settings and locales of data collection, noting the time periods of recruitment and data collection.*
Outcomes	*Describe how you pre-defined primary and secondary outcome measures and how you assessed these measures.*
