# Supplementary material for: Genetic screening identifies a SUMO protease dynamically maintaining centromeric chromatin
Source: Nat Commun. 2020 Jan 24;11:501. doi: 10.1038/s41467-019-14276-x (PMC6981222; doi:10.1038/s41467-019-14276-x)
Supplement: Supplementary file 4 — Description of Additional Supplementary Files [file 41467_2019_14276_MOESM4_ESM.pdf]

Description of Additional Supplementary Files

File Name: Supplementary Data 1

Description: List of all 2172 siRNA sequences with their respective gene targets and ENSEMBL IDs.

File Name: Supplementary Data 2

Description: Ranking of all siRNA targets according to their impact on CENP-A maintenance in SNAP pulse-chase screen. Ranking is based on Fold difference “t test positions estimates”. Scores for Significance “t test positions p value” with respective sign code, Z scores and number of objects (cell nuclei) are also listed. The descriptors are defined in methods.

File Name: Supplementary Data 3

Description: Ranking of all siRNA targets according to their impact on CENP-A assembly in SNAP quench-chase-pulse screen. Ranking parameters are as in Supplementary Data 2 and are defined in the methods.

File Name: Source Data file 1

Description: Tabulated data file listing all image quantification data for histogram plots in Figures 2-5 and Supplemental Figures 2-4.

File Name: Source Data file 2

Description: Raw images for all immunoblots presented in Figures 4-6 and Supplemental Figures 2 and 4.
