# Supplementary material for: Genetic screening identifies a SUMO protease dynamically maintaining centromeric chromatin
Source: Nat Commun. 2020 Jan 24;11:501. doi: 10.1038/s41467-019-14276-x (PMC6981222; doi:10.1038/s41467-019-14276-x)

Note: Each pair of images consists of two channels of the same blot

Fig. 4D

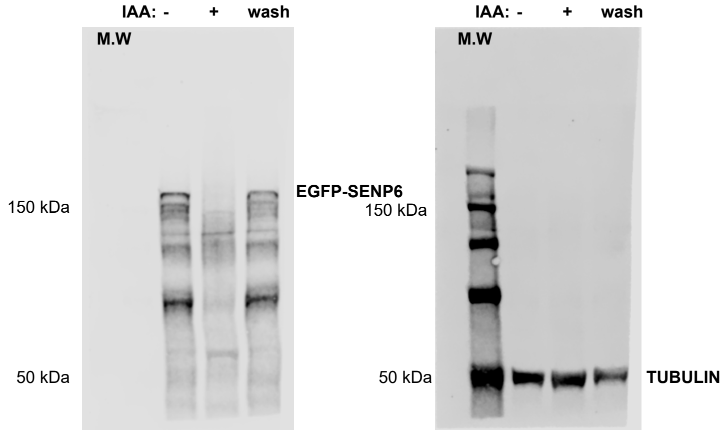

Fig. 5E (top)

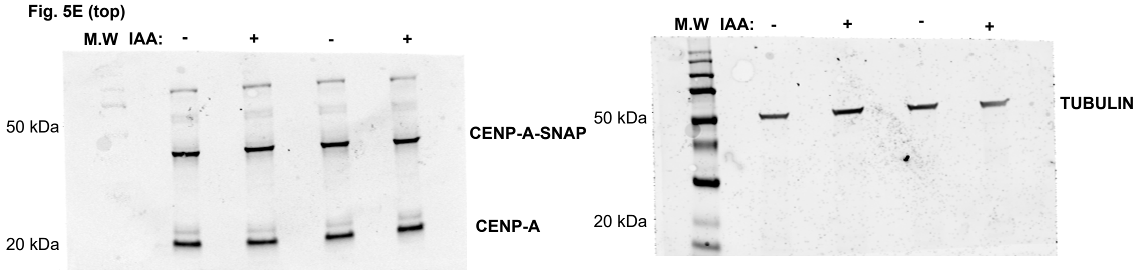

Fig. 5E (bottom)

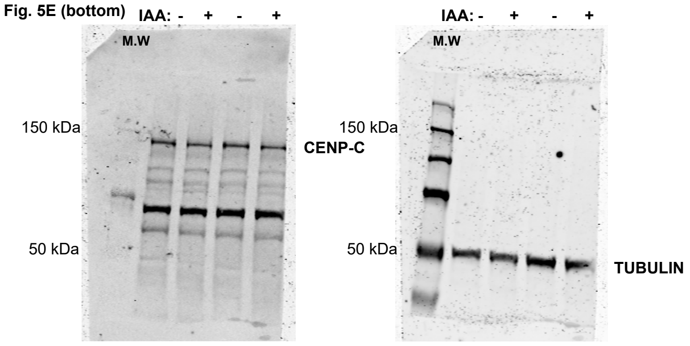

Fig. S2A

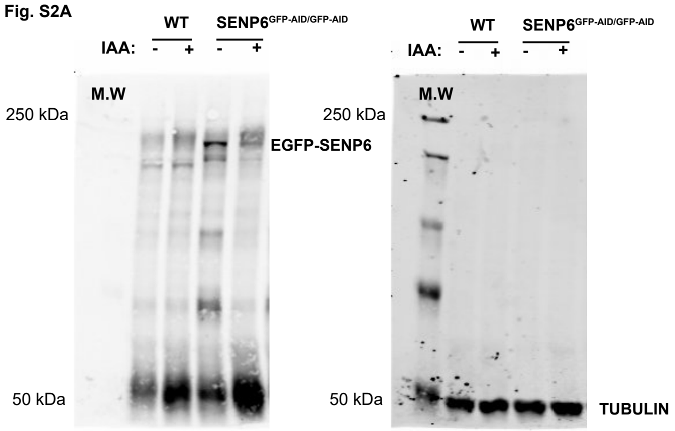

Fig. S4C (top)

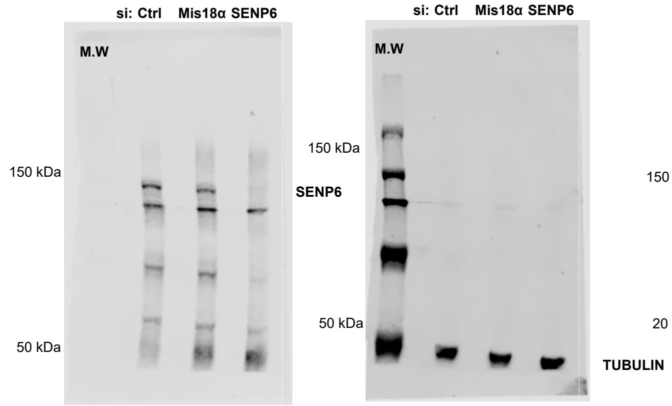

Fig. S4C (middle)

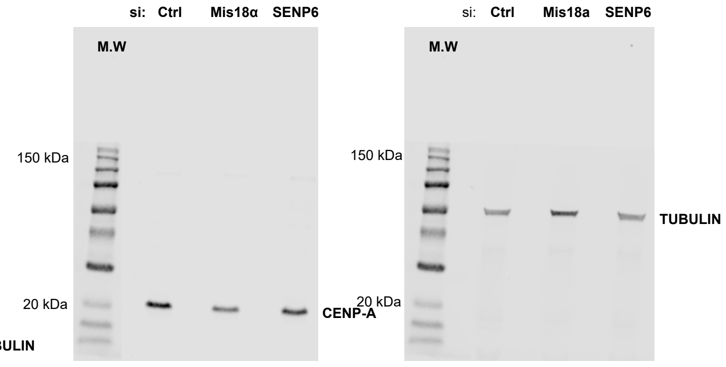

Fig. S4C (bottom)

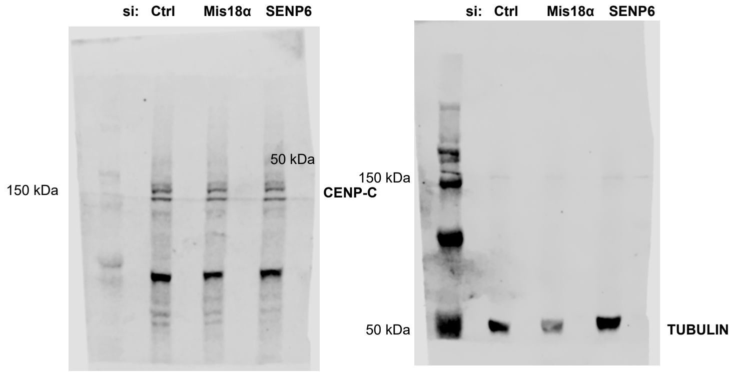

Note: Each pair of images consists of two channels of the same blot

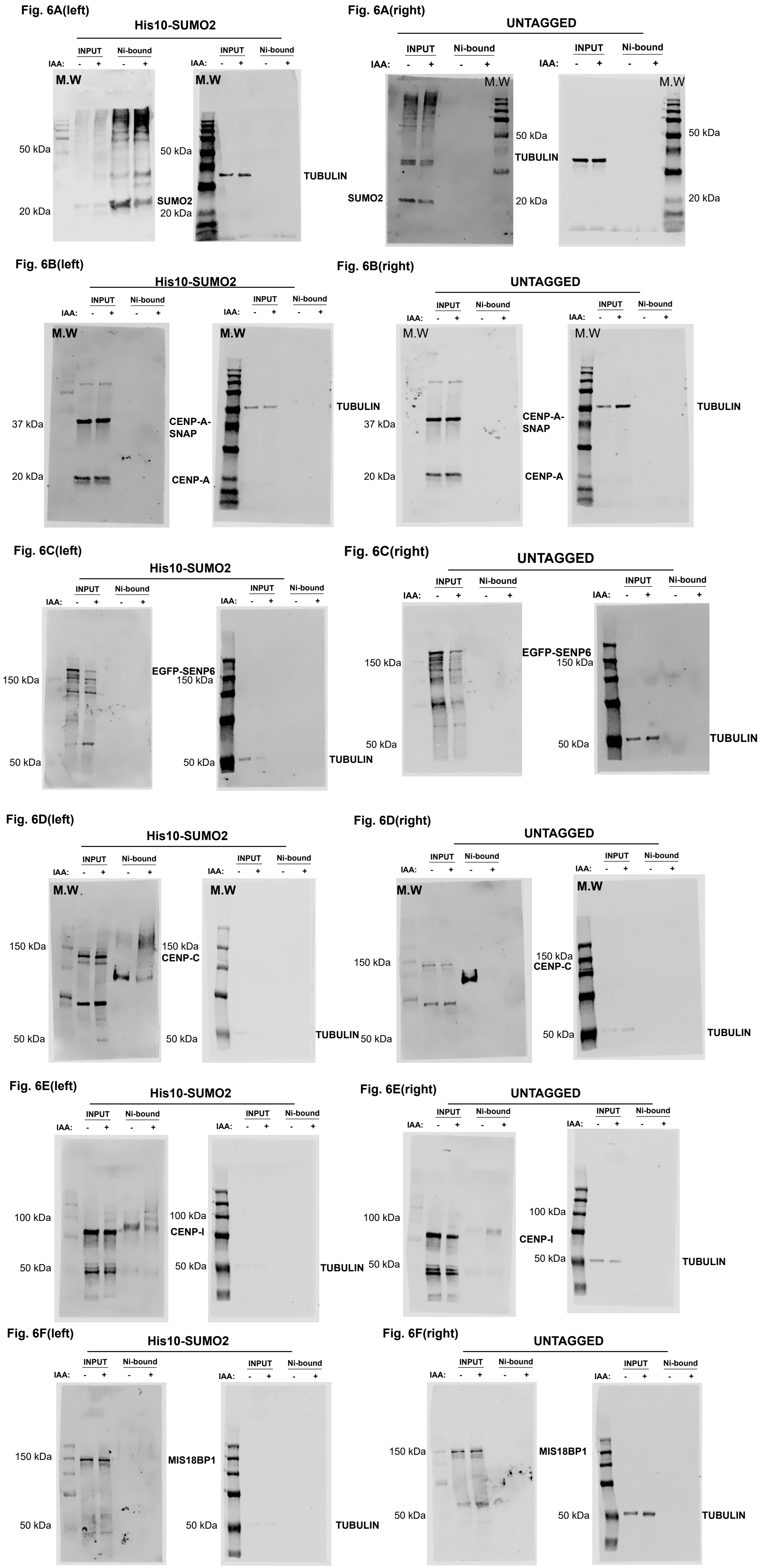

Supplement: Supplementary file 9 — Source Data file 2 [file 41467_2019_14276_MOESM9_ESM.pdf]
